# Survey of artemisinin production by diverse *Artemisia *species in northern Pakistan

**DOI:** 10.1186/1475-2875-9-310

**Published:** 2010-11-04

**Authors:** Abdul Mannan, Ibrar Ahmed, Waheed Arshad, Muhammad F Asim, Rizwana A Qureshi, Izhar Hussain, Bushra Mirza

**Affiliations:** 1 Department of Biochemistry, Quaid-i-Azam University, Islamabad, Pakistan; 2 Department of Plant Sciences, Quaid-i-Azam University, Islamabad, Pakistan; 3 Department of Pharmaceutical Sciences, COMSATS Institute of Information Technology, Abbottabad, Pakistan; 4 Institute of Molecular Biosciences, Massey University, Palmerston North, New Zealand

## Abstract

**Background:**

Artemisinin is the current drug of choice for treatment of malaria and a number of other diseases. It is obtained from the annual herb, *Artemisia annua *and some microbial sources by genetic engineering. There is a great concern that the artemisinin production at current rate will not meet the increasing demand by the pharmaceutical industry, so looking for additional sources is imperative.

**Methods:**

In current study, artemisinin concentration was analysed and compared in the flowers, leaves, roots and stems of *Artemisia annua *and 14 other *Artemisia *species including two varieties each for *Artemisia roxburghiana *and *Artemisia dracunculus *using high performance liquid chromatography (HPLC).

**Results:**

The highest artemisinin concentration was detected in the leaves (0.44 ± 0.03%) and flowers (0.42 ± 0.03%) of *A. annua*, followed by the flowers (0.34 ± .02%) of *A. bushriences *and leaves (0.27 ± 0%) of *A. dracunculus var dracunculus*. The average concentration of artemisinin varied in the order of flowers > leaves > stems > roots.

**Conclusion:**

This study identifies twelve novel plant sources of artemisinin, which may be helpful for pharmaceutical production of artemisinin. This is the first report of quantitative comparison of artemisinin among a large number of *Artemisia *species.

## Background

Despite advances in medical sciences, malaria is still a global health problem causing a death toll of approximately one million per annum [1]. More than one billion people live in areas with high malarial risk [2]. There were 243 million cases of malaria in 2008, causing 863,000 deaths, mostly among children in Africa [3], where malaria accounts for 20% of all childhood deaths [4]. This has resulted in enormous pressure on economy of the countries with high disease rates, causing as much as 1.3% reduction in GDP, up to 40% of public health expenditures, 30% to 50% of inpatient hospital admission, and 60% outpatient visits to health clinics [4]. Artemisinin combination therapy (ACT) is currently the most effective means to treat and reduce the transmission rate of malaria [5]. The World Health Organization (WHO) recommends ACT as first-line treatment for uncomplicated malaria caused by *Plasmodium falciparum *[6]. Artemisinin has also been demonstrated to be effective against some other parasites including *Leishmania *[7], *Schistosoma *[8], *Toxoplasma *[9] and *Trypanomosa *[10]. It has also antiviral activities [11] and can be used in treatment of hepatitis B [12] and a range of cancer cell lines, including breast cancer, human leukaemia, colon, and small-cell lung carcinomas [13]. Furthermore, artemisinin may be especially effective in treating drug resistant cancers [14,15]. Another important role of artemisinin is its allelopathic activity; it is a potent plant inhibitor with potential as a natural herbicide [16]. Artemisinin has been considered as a relatively safe drug with no obvious adverse reactions or serious side effects, even for pregnant women [17]. A recent study [18], however, outlines potential toxic effects of artemisinin when used for long periods and offers suggestions for safe use.

For a pharmaceutical industry supplying over 100 million treatments per annum [2], there is a growing concern that the artemisinin supply chain will be unable to meet future requirements [19,20]. Access of patients to artemisinin-based combination therapy was inadequate in all countries surveyed during 2007 and 2008 [3]. Microbial-based systems to synthesize artemisinin precursors for chemical conversion have been reported [21-24]. However, agricultural production is likely to remain the primary source of supply [19]. Artemisinin is a sesquiterpenoid, produced in glandular trichomes of *Artemisia annua *[25]. *Artemisia annua*, a member of the Asteraceae family, is endemic to China and has been used there for over two thousand years to treat malaria [26]. Other species of *Artemisia *have been screened as potential new sources for agricultural production. Artemisinin has been reported in *Artemisia apiacea *and *Artemisia lancea *[26], *Artemisia cina *[27] and in aerial parts of *Artemisia sieberi *[28]. Presence of artemisinin has also been reported in *Artemisia absinthium *[29], *Artemisia dubia *and *Artemisia indica *[30] growing in Pakistan.

Twenty five species of *Artemisia *are known to exist in Pakistan [31] and most of them are found in northern frontier regions, in foothills and ranges of the Karakoram, Himalaya and Hindu Kush mountains, in districts Gilgit, Chitral, Skardu, Swat, Rawalpindi, Abbottabad and Kashmir (personal observations). Many of these species have a long history of use as folk therapeutic plants [32]. Purpose of the current study was to explore potential new sources of artemisinin, and compare artemisinin concentration in different parts of various *Artemisia *species found in northern areas of Pakistan. The study reports 12 new potential sources of artemisinin identified first time as well as and their artemisinin concentration compared in flowers, leaves, stems and roots.

## Methods

**Figure 1:**
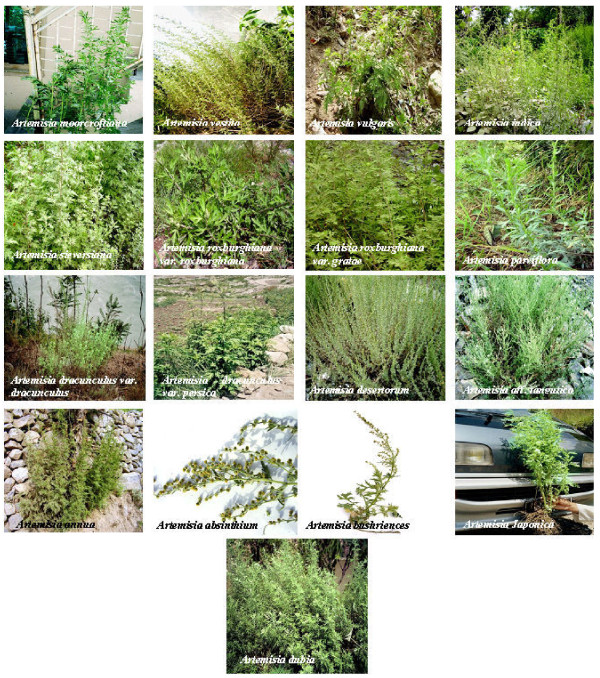
**Field pictures of seventeen Artemisia species at the time of collection**.

**Figure 2:**
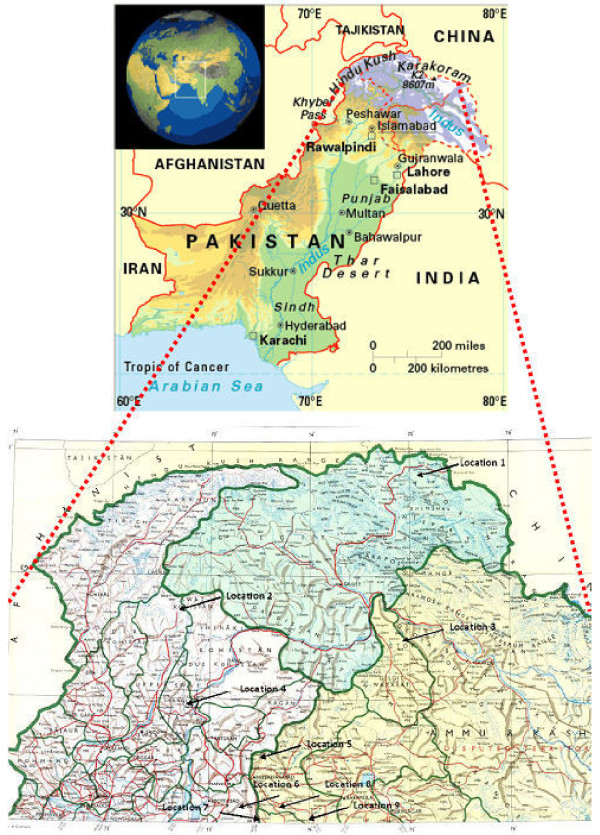
**Geographical map of Northern Pakistan showing different locations of collection of Artemisia species**.

In total, fifteen indigenous *Artemisia *species including two varieties each for two species (*Artemisia roxburghiana *and *Artemisia dracunculus*; Figure 1) were collected in summer 2007 from various Himalayan, Karakoram and Hindu Kush ranges of Northern Pakistan (Figure 2). All seventeen species [33] were harvested and collected at flowering stage. Their population was found dense in the locations mentioned in Table 1. The complete distribution patterns of individual species will be covered elsewhere. These species were identified in Herbarium of Islamabad, Pakistan, by following the description in Flora of Pakistan [31] and comparing with already identified herbarium sheets of same *Artemisia *species preserved in the herbarium. These species were given the herbarium numbers and were deposited in the herbarium collection. These *Artemisia *species were screened to measure the artemisinin concentration in their four parts including flowers, leaves, stems and roots.

**Table 1:** *Artemisia *species collected at flowering stage from different hilly locations of Northern Pakistan

**S. No**.	Species	**Location No**.	Location name	Sampling Point
1	*A. moorcroftiana*	2	Kalam, Swat	Saeed Abad
2	*A. vestita*	6	Galyat	Donga Gali
3	*A. vulgaris*	2	Kalam, Swat	Gatal
4	*A. indica*	4	Shangla, Swat	Topseen
5	*A. sieversiana*	1	Soost, Northern Areas	Aaeen Abad
6	*A. roxburghiana var roxburghiana*	8	Rawalakot, Azad Kashmir	Mujahid Abad
7	*A. roxburghiana var gratae*	7	Murree, Rawalpindi	Bhurban
8	*A. parviflora*	8	Rawalakot, Azad Kashmir	Tararh Khal
9	*A. dracunculus var dracunculus*	9	Abbass Pur, Azad Kashmir	Abbass Pur
10	*A. dracunculus var persica*	8	Rawalakot, Azad Kashmir	Rawalakot
11	*A. desertorum*	4	Shangla, Swat	Shangla Hill Top
12	*A. aff. Tangutica*	5	Shugran, Mansehra	Payee Base
13	*A. annua*	3	Astore, Northern Areas	Kachar Abad
14	*A. absinthium*	3	Astore, Northern Areas	Chilam Choki
15	*A. bushriences*	1	Soost, Northern Areas	Aaeen Abad
16	*A. japonica*	6	Galyat	Donga Gali
17	*A. dubia*	6	Galyat	Donga Gali

For analysis and quantification of artemisinin, five plants per *Artemisia *species were harvested at any given location. Artemisinin was analysed by HPLC in triplicate from each part of each of these five plants (i.e. a total of 15 samples for each individual part per species). Artemisinin was extracted from different parts i.e. flowers, leaves, stems and roots of all *Artemisia *species following a reported methodology [34] with some modifications as follows: one gram each of fresh floral, leaf, stem and root parts of each *Artemisia *species was taken and placed in oven at 60°C for three days. Floral parts included complete inflorescence, leaves included whole leaves with short stalks inclusive and plucked from upper surface of the plants, which were maximally exposed to light, stems included green slender branches connected to the leaves, while roots comprised mostly of parts close to the root tips. Their dry weights were measured; the tissues were grinded with mortar and pestle and put in 5 ml of HPLC grade toluene (Sigma) to make a homogenous mixture. These mixtures were placed in sonicator (Elma™, Germany) for 30 min. During sonication, artemisinin was released into toluene, which was separated from cellular debris by centrifugation at 2000 ×g and -8ºC for 20 minutes (Eppendorf centrifuge, model 5810R). Supernatant was decanted and saved in dram vials. Pellets were re-suspended in 5 ml toluene, vortexed, sonicated again for 30 minutes, centrifuged and decanted as above. Pellets were discarded, both supernatants were pooled; extracts for each replicate were air dried before storing at -20ºC for further analysis.

The dried extracts and dried dilutions of standard artemisinin were converted to Q260 derivative of artemisinin as follows: each extract was solubilized in 400 μl of methanol (Sigma) and 1600 μl of 0.2% (w/v) NaOH,; then hydrolyzed for 45 min at 50ºC. The reaction was stopped by adding 1600 μl of 0.2 M acetic acid (Merck) and placing the test tube on ice. To make a final volume of 4 ml, 400 μl of methanol was added. Samples at this stage contained Q260 derivative of artemisinin, and were filtered through 0.45 μm filter before injecting into HPLC for analysis. All samples were hydrolyzed just before use.

For HPLC analysis, mobile phase was prepared by combining 45% (v/v) methanol (Sigma) and 55% 0.01 M sodium phosphate buffer (pH 7.0). All samples were analysed with a Zorbax SB C18 column (150 × 4.6 mm × 5 μm; Agilent Technologies USA). Flow of the mobile phase through column (stationary phase) was optimized to 1 ml/min. Artemisinin was detected by using Diode Array Detector (G1315B-DAD) at 260 nm absorbance and its retention time was 12 min. Artemisinin concentration was calculated as a percentage of dry weight, average values were calculated for each part, and data were analysed using ANOVA and LSD.

## Results

**Figure 3:**
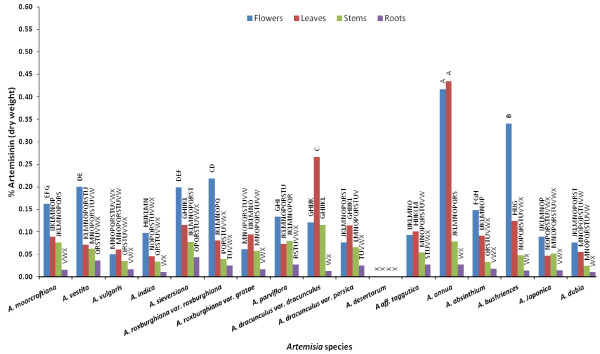
**Comparative concentration of artemisinin in flowers, leaves, stem and roots of all seventeen Artemisia species (Bar represents the mean values of artemisinin in each treatment and the alphabets above represents the LSD ranking of these values at α = 0.05)**.

**Figure 4:**
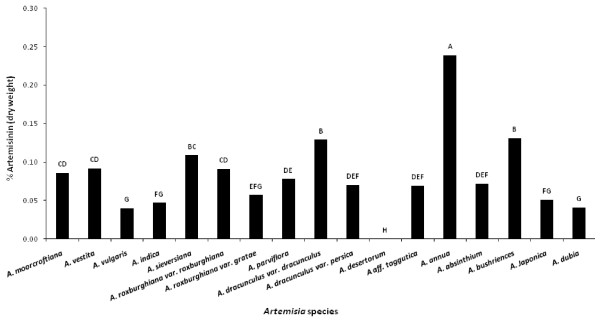
**Comparison of whole plant parts (flowers, leaves, stem and roots) artemisinin concentration among all seventeen *Artemisia *species (Bar represents the mean values of artemisinin in each treatment and the alphabets above represents the LSD ranking of these values at α = 0.05)**.

Amongst all *Artemisia *species studied, the highest artemisinin concentration was found in leaves of *Artemisia annua *(0.44 ± 0.03%), followed by flowers of *A. annua *(0.42 ± 0.03%), flowers of *Artemisia bushriences *(0.34 ± 0.02%), and leaves of *Artemisia dracunculus var dracunculus *(0.27 ± 0%), as shown in Figure 3 &4.

*Artemisia annua *showed highest and almost equal artemisinin concentration in flowers and leaves. Equal levels of artemisinin were also detected in the *Artemisia vulgaris *and *Artemisia aff tangutica *leaves and flowers. Ten species (*Artemisia moorcroftiana, Artemisia vestita, Artemisia indica, Artemisia sieversiana, Artemisia roxburghiana var roxburghiana, Artemisia parviflora, Artemisia absinthium, Artemisia bushriences, Artemisia japonica *and *Artemisia dubia*) showed higher artemisinin concentration in their flowers, while two species (*Artemisia roxburghiana var. gratae, Artemisia dracunculus var. dracunculus *&*var. persica*) showed higher artemisinin in their leaves. No artemisinin was found in all four tissues of *Artemisia desertorum*.

The overall comparison of artemisinin concentration in individual parts across all species showed that among the flowers, highest artemisinin concentration was found in *A. annua *(0.42 ± 0.03%), followed by *A. bushriences *(0.34 ± 0.02%), and *A. roxburghiana var roxburghiana *(0.23 ± 0.01%), as shown in Figure 3. Among leaves, the highest artemisinin was found in *A. annua *(0.44 ± 0.03%), followed by *A. dracunculus var dracunculus *(0.27 ± 0%). In stems, the highest artemisinin was in *A. dracunculus var dracunculus *(0.12 ± 0.01%) followed by *A. parviflora*, *A. moorcroftiana, A. sieversiana *and *A. annua *(0.8 ± 0%). Among roots, the highest artemisinin concentration was found in *A. vestita *and *A. sieversiana *(0.04 ± 0%). The current study reports artemisinin in 12 species of *Artemisia *for the first time. Table 2 lists all *Artemisia *species which have been studied with respect to artemisinin yield up to now. It can be anticipated, however, that some variation in artemisinin concentration in previously reported work may be possible due to differences in methods of artemisinin extraction.

**Table 2:** Summary of previous reports from different parts of the world showing percentage of artemisinin in different parts of *Artemisia *species

**S. No**.	Artemisia species	Part used	Artemisinin Percentage	Country of origin	Reference
1	*A. annua*	Leaves	0.60	China	[64]
2	*A. annua*	Leaves	0.10	Argentina	[61]
3	*A. annua*	Leaves	0.02	Germany	[49]
4	*A. annua*	Leaves	0.79, 0.14	China	[44]
5	*A. annua*	Leaves	0.21	USA	[44]
6	*A. annua*	leaves	0.24	Spain	[53]
7	*A. annua*	Leaves	1.07	China	[53]
8	*A. annua*	Leaves	1.38	Switzerland	[53]
9	*A. annua*	Leaves	0.86	Vietnam	[65]
10	*A. annua*	Leaves	0.46	Vietnam	[66]
11	*A. annua*	Leaves	0.52	Netherlands	[66]
12	*A. cina*	Shoots	0.0006	Indonesia	[27]
13	*A. apiacea*	Leaves	Not shown	China	[26]
14	*A. lancea*	Leaves	Not shown	China	[26]
15	*A. sieberi*.	Aerial Parts	0.2	Iran	[28]
16	*A. absinthium*	Leaves	0.022	Pakistan	[29]
17	*A. dubia*	Roots	Variable	Pakistan	[30]
18	*A. indica*	Roots	Variable	Pakistan	[30]

## Discussion

Many natural products in plants are multifunctional molecules that protect them from infections of bacteria, viruses, and other microorganisms, or from herbivores such as insects, worms, and mammals [35]. Artemisinin has been shown to be active against diverse plant pathogenic fungi including *Gaeumannomyces graminis var. tritici, Rhizoctonia cerealis, Gerlachia nivalis*, and *Verticillium dahliae *[36].

Dried *Artemisia *parts have been used in many studies [37-39] for qualitative as well as quantitative analysis of artemisinin; artemisinin analysis in current study was also carried out on dried parts. Artemisinin has previously been extracted with n-hexane [38], toluene [40], chloroform [41] and petroleum ether [42] and with extraction times ranging from a few minutes [43] to several hours [44]. In current study, extraction of artemisinin was carried out in toluene, as it is currently the most widely used solvent [30,34,39].

Many analytical procedures to identify and quantify artemisinin have been developed during the last three decades. These include thin layer chromatography (TLC) [42], high performance liquid chromatography with UV detection (HPLC-UV) [45,46], gas chromatography coupled to mass spectrometry (GC-MS) [41], capillary electrophoresis coupled to UV detector (CE-UV) [47] and enzyme-linked immunosorbent assay (ELIZA) [48]. Artemisinin analysis in this study was carried out by using HPLC-UV method for rapid, accurate and cost effective detection and quantification of artemisinin. This is currently the most commonly used method for artemisinin assays [34,39,45].

A prominent feature of current research work was to screen out maximum available *Artemisia *species for the presence of artemisinin by using same procedure of extraction (using toluene), same equipment for analysis (HPLC-UV) and same type of plant tissues (dried plant tissues), in four different parts (flowers, leaves, stems, roots) of plants growing at same stage (flowering stage).

Comparison of artemisinin yield at different stages of plant development shows a positive correlation between the plant age and artemisinin yield [49]. For *A. annua*, the highest artemisinin concentration has been reported in leaves and flowers during full bloom stage, in comparison to the pre- and post-flowering stages [50]. Some reports show 0.54% and 0.45% artemisinin concentration in leaves and flowers of *A. annua *respectively [50]; concentration ranging from 0.01% to 0.80% in leaves and flowers of *A. annua *[51], 0.46% and 0.52% in leaves of *A. annua *from Vietnam and Netherlands respectively [52], 0.24% in *A. annua *dry leaves [53], while 0.42% in some individual U.S. strains at the full flowering stage [54]. The present findings suggest similar results; Figure 3 shows that the highest and almost equal artemisinin concentration was detected in leaves (0.44 ± 0.03%) and flowers (0.42 ± 0.03%) of *A. annua*. An important finding is the presence of appreciable concentration of artemisinin in either leaves or flowers of six *Artemisia *species including *A. vestita, A. sieversiana, A. roxburghiana var roxburghiana, A. dracunculus var dracunculus, A. annua *and *A. bushriences*, in the range from 0.20% to 0.44% (Figure 3). Some reports had previously shown the presence of artemisinin in *Artemisia *species other than *A. annua*, but the artemisinin concentrations were either negligible [27] or low [28], as shown in Table 2.

Flowers and leaves of ten *Artemisia *species including *A. moorcroftiana*, *A. vulgaris*, *A. indica*, *A. roxburghiana var gratae*, *A. parviflora*, *A. dracunculus var persica*, *A. aff tangutica*, *A. absinthium*, *A. japonica*, and *A. dubia *were found to contain artemisinin in the range of 0.05% to 0.15%, as presented in Figure 3. Six of these ten species contain more artemisinin in flowers than that in leaves, which is in agreement with a prior study [55], reporting that artemisinin content of inflorescence in bud stage was not higher than in leaves, but artemisinin in flowers at full bloom was 4 to 11 fold higher than in leaves of *A. annua*. Generally, leaves are much more abundant than flowers in species studied, and leaves can provide more artemisinin on average per plant than flowers. According to a report [44] leaves contribute the major dry matter for artemisinin extraction, and 89% of total plant artemisinin comes from leaves. This is the reason of commercial use of *A. annua *leaves for extraction of artemisinin in China, Vietnam and other countries of the world [56]. Furthermore, upper portion of leaves or aerial parts of *A. annua *yield more artemisinin than lower portion of leaves.

Artemisinin concentration in roots of all *Artemisia *species in current study was non-significant (Figure 3). These findings are in agreement with previous studies [34,57] showing very low artemisinin concentration (0.003%) in the roots of *A. annua*. Artemisinin was not found in roots of *A. annua *in some studies [41,58,59]. The overall results in the current study suggest presence of high artemisinin concentration in either flowers or leaves and low or zero artemisinin concentration in stems and roots (Figure 3), which is in congruence with a previous report [44], showing artemisinin mostly in leaves and inflorescence of *A. annua*, while low levels in stem and none in the roots (Table 2).

Artemisinin is synthesized in specialized capitate glandular trichomes of *A. annua *[60]. Another study [43] shows that artemisinin is stored in sub-cuticular space of capitate glandular trichomes of *Artemisia *species. Artemisinin accumulates in flowers, leaves, young green stems, buds and seeds where glandular trichomes are present [61,62]. Artemisinin and the characteristic aroma of *Artemisia *plants were absent in foliar tissues of a biotype of *A. annua *lacking glandular trichomes; no ultra-structural differences were found in the mesophyll cells of above biotype with normal biotypes [60]. The current study was focused on flowers, leaves, stems and roots of *Artemisia *species. Artemisinin concentration in these tissues correlated with the glandular trichome studies of *A. annua*. The association of peak artemisinin with flowering is related to the abundance of glandular trichomes in inflorescence, particularly florets and receptacle [57]. Absence of trichomes and artemisinin could be due to different genetic and environmental factors. Genetic variations have been reported for the plants that retain glands, but lack artemisinin [63]. A recent publication of the genetic map of *A. annua *identifying loci which affect artemisinin production [2] will foster future research into *Artemisia *genomics.

## Conclusion

This study reported potential new plant sources of artemisinin, in addition to *A. annua*. These *Artemisia *species are abundant in distribution in northern Pakistan. The description of their full geographic distribution is beyond the scope of this study. Some of these species yield comparable amounts of artemisinin to that of *A. annua *(Table 2), and can be screened further for commercial extraction on artemisinin, although most of these are being used as folk therapeutic plants for various purposes [32].

## Competing interests

The authors declare that they have no competing interests.

## Authors' contributions

AM, IA and WA contributed in geographical survey, plant collection and processing; AM and RAQ carried out species identification; AM and MFA carried out HPLC assays; AM, IH and BM contributed in results analysis and study design; AM, IA and BM prepared the manuscript, BM supervised the study. All authors read and approved the final manuscript.
